# Applying Innovative Qualitative Methods to Understand the Perspective of Vulnerable and Diverse Populations

**DOI:** 10.1093/geroni/igaa057.1863

**Published:** 2020-12-16

**Authors:** Martina Roes, Chaya Koren

## Abstract

The application of a variety of innovative qualitative research methods and analysis as well as the possibilities it offers for the selected population to share their personal experience is the main focus of this symposium. We present the relationships between design, methodological approaches and themes of interest for the people who participate in research. The presenters used designs such as ethnographic field research or phenomenological designs. The used photos taken and analyzed by the participants; using photo-voice and tabletop exercise. All speakers will present their designs and methods linked to a specific research theme. Using examples of recent and highly innovative research practices which meaningfully challenge taken-for-granted assumptions in social science and care research, to open new ground for other ways of thinking about doing research in these fields. Goal for the discussion is a critical reflection of the designs and methods used and to provide take away messages

